# Data on prediction of geological characteristics during shield tunnelling in mixed soil and rock ground

**DOI:** 10.1016/j.dib.2022.108726

**Published:** 2022-11-04

**Authors:** Tao Yan

**Affiliations:** a MOE Key Laboratory of Intelligent Manufacturing Technology, Department of Civil and Environmental Engineering, College of Engineering, Shantou University, Shantou, Guangdong 515063, China; b Discipline of Civil and Infrastructure Engineering, School of Engineering, Royal Melbourne Institute of Technology (RMIT), Victoria 3001, Australia

**Keywords:** Shield operational parameters, Geological characteristics, Identification and cluster, Stacking algorithm

## Abstract

This data presented in this article pertain to measured data obtained from earth pressure balance (EPB) shield tunelling of Guangzhou-Foshan intercity railway project. The measured data consists of geological characteristics and the main shield parameters in each lining ring during shield tunnelling. The distribution of raw data was displayed, and the geological characteristics via field record were compared to the prediction results of improved stacking method. The value of the database is consideration of the relationship between shield operational parameters and geological characteristics encountered in the shield tunnelling area, including formations with soft soil, majority of soft soil, and majority of hard rock. The raw data was standardized and processed to low dimensional data by principal component analysis, which can be better used in geological characteristics classification. The presented data are applied to identify the geological characteristics in the article titled “Prediction of geological characteristics from shield operational parameters by integrating grid search and K-fold cross validation into stacking classification algorithm”.

## Specifications Table

**Table:** 

Subject area	Engineering Geology
More specific subject area	Geotechnical engineering, shield tunnelling
Type of data	Tables, Figures
How data was acquired	Data were recorded by sensors of shield machine and mathematical calculation
Data format	Original and analyzed
Parameters for data collection	Shield parameters in each lining ring were collected by sensors of shield machine. Geological characteristics from the site were recorded by site engineers.
Description of data collection	The shield parameters and geological characteristics were necessary to be analyzed in each segment to ensure the safety of shield tunnelling.
Data source location	Guangzhou City, China
Data accessibility	Data provided in this article and supplementary material in Mendeley Data. doi: https://doi.org/10.17632/cjwbjttf88.1.
Related research article	T. Yan, S.L. Shen, A. Zhou, Prediction of geological characteristics from shield operational parameters by integrating grid search and K-fold cross validation into stacking classification algorithm, J. Rock Mech. Geotech. Eng. 14 (2022) 1292-1303. https://doi.org/10.1016/j.jrmge.2022.03.002. [1]

## Value of the Data

• The data of shield operational parameters can be used to analyze the relationship with the geological characteristics during shield tunnelling [2,3].
• The data of geological characteristics in each lining ring can be applied to ensure the safety of shield tunnelling process and arrange the schedule of construction [4,5].
• The data can help other researchers focus on the efficiency of shield tunnelling and the project's cost [6,7]. Additionally, scholars can evaluate the performance and risk during shield tunnelling in soil-rock ground [8], [9], [10].
• The steps of geological characteristics prediction can help researchers understand the process and application of the stacking classification algorithm integrated with grid search (SCA-GS).

## 1 Data Description

**Fig. 1:**
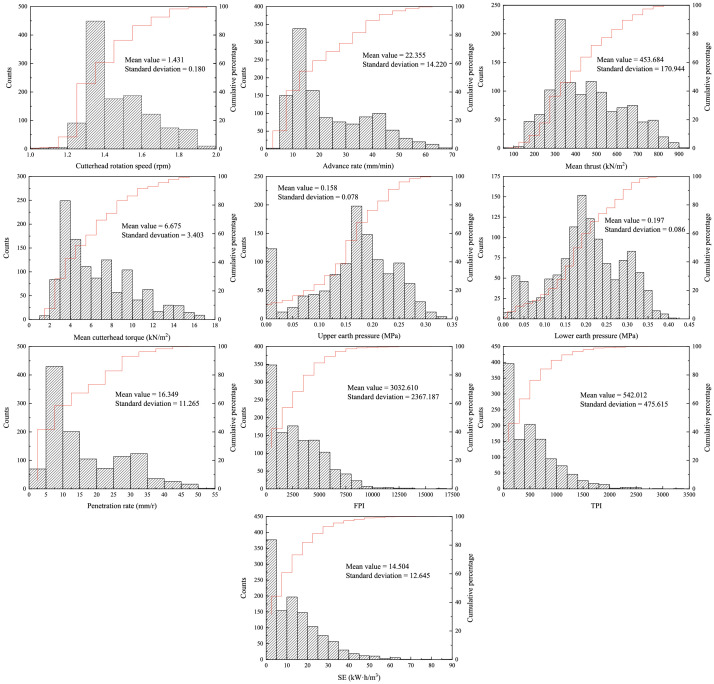
Distribution of original data.

In this article, the database consists of field-measured shield parameters by sensors and types of geological characteristics recorded by shield operators. The operational parameters were collected from acquisition system in shield machine. Additionally, the transformed parameters were also considered as input data shown in this article. Fig. 1 indicates the distribution of original input data, which can help readers better understand the data structure. The original data consists of 6 shield parameters: cutterhead rotation speed (CRS), advance rate (AR), mean thrust (MF), mean cutterhead torque (MT), upper earth pressure (UEP), and lower earth pressure (LEP) and four transformed factors: penetration rate (PR), torque penetration index (TPI), specific energy (SE), and field penetration index (FPI) [11], [12], [13]. The dimension of original data was reduced to 6 principal components through principal component analysis (PCA). The geological characteristics were predicted using an improved stacking classification algorithm with original data and the data with reducing dimensions using PCA (PCA data). Then, the geological characteristics can be classified by SCA-GS algorithm.

**Fig. 2:**
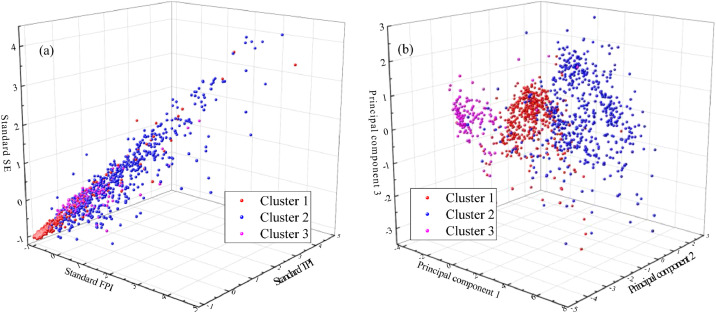
Visualization of geological characteristics in the record using: (a) original data; (b) PCA data.

Fig. 2 shows the geological characteristics in record after shield tunnelling. The original data (standard FPI, standard TPI, and standard SE in Fig. 2a) and PCA data (principal components 1, 2, and 3) were employed to display the 3D feature space to show the distribution of geological characteristics.

## 2 Experimental Design, Materials and Methods

The shield parameters are the reaction of the geological characteristics variety. The geological conditions are always continued in a construction site. Therefore, the shield parameters can be clustered as factors to evaluate the types of geological characteristics [14], [15], [16].

In this article, six shield parameters and four transformed parameters were used to analyze the geological characteristics [17]. The correlation of these factors and geological characteristics was assessed by Spearman correlation coefficient (SCC) in Table 1. The result of Spearman correlation coefficient shows that PR and AR are negatively correlated with the geological characteristics. On the other hand, the TPI, SE, and FPI are the most positively correlated with geological characteristics. Therefore, the TPI, SE, and FPI can be better used to show the results of geological characteristics identification.

**Table 1:** Spearman correlation coefficient for shield parameters and geological characteristics.

	CRS	AR	MF	MT	UEP	LEP	PR	FPI	TPI	SE	GC
CRS	1	-0.433^a^	0.056	0.103^a^	0.056	0.083^a^	-0.571^a^	0.431^a^	0.412^a^	0.412^a^	0.583^a^
AR	-0.433^a^	1	-0.301^a^	-0.394^a^	-0.057^b^	-0.151^a^	0.984^a^	-0.879^a^	-0.858^a^	-0.857^a^	-0.694^a^
MF	0.056	-0.301^a^	1	0.780^a^	0.803^a^	0.871^a^	-0.257^a^	0.690^a^	0.602^a^	0.606^a^	0.139^a^
MT	0.103^a^	-0.394^a^	0.780^a^	1	0.517^a^	0.635^a^	-0.361^a^	0.647^a^	0.784^a^	0.786^a^	0.276^a^
UEP	0.056	-0.057^b^	0.803^a^	0.517^a^	1	0.929^a^	-0.035	0.422^a^	0.314^a^	0.317^a^	0.023
LEP	0.083^a^	-0.151^a^	0.871^a^	0.635^a^	0.929^a^	1	-0.125^a^	0.520^a^	0.434^a^	0.437^a^	0.056
PR	-0.571^a^	0.984^a^	-0.257^a^	-0.361^a^	-0.035	-0.125^a^	1	-0.864^a^	-0.846^a^	-0.84^a^	-0.740^a^
FPI	0.431^a^	-0.879^a^	0.690^a^	0.647^a^	0.422^a^	0.520^a^	-0.864^a^	1	0.931^a^	0.932^a^	0.595^a^
TPI	0.412^a^	-0.858^a^	0.602^a^	0.784^a^	0.314^a^	0.434^a^	-0.846^a^	0.931^a^	1	1.000^a^	0.616^a^
SE	0.412^a^	-0.857^a^	0.606^a^	0.786^a^	0.317^a^	0.437^a^	-0.84^a^	0.932^a^	1.000^a^	1	0.615^a^
GC	0.583^a^	-0.694^a^	0.139^a^	0.276^a^	0.023	0.056	-0.740^a^	0.595^a^	0.616^a^	0.615^a^	1

a Correlation significance at the 0.01 level;

b Correlation significance at the 0.05 level (double tail)

Table 2 presents the steps of geological characteristics prediction using SCA-GS. Before conducting the SCA-GS, the original data was standardized and processed by PCA. The results of PCA process were considered as input data with *k* dimensions (*D*). Then, 80% of the database was randomly selected as a training set, and the remaining 20% of the database was considered as test set [18]. The dataset, primary classifiers, and meta-classifier were input in the SCA-GS model. The selected hyper-parameters of each primary classifier were set pairs using GS. Next, the primary classifiers with paired hyper-parameters were trained using training set and evaluated by K-CV. Then, the best models and corresponding hyper-parameters with the highest accuracy were selected to integrate the stacking algorithm. The results of primary classifiers can be employed to train the meta-classifier. Finally, the classification results were obtained using the meta-classifier on the test set. More detailed data processing, classification process can be found in companion article [1].

**Table 2:** Steps of geological characteristics prediction using SCA-GS.

**Algorithm:** The calculation process of SCA-GS for geological characteristics prediction [1]
**Data preparation:** dataset with m dimensions was standardized and processed to *k* dimensions using PCA. There are N pieces in original data {*x^n^, n* = 1, 2, …, *N*}. The original dataset contains ten identification factors. The results of PCA process were considered as input data with *k* dimensions (*D*). 1. **Input:** training set and test set, primary classifiers: C_1_, C_2_, …, C*_I_*, and meta-classifier: C. 2. Set pairs for selected hyper-parameters of each classifier using GS. **for***i*= 1, 2, …, *I***do** Param*_i_*={*p*_1_: (*a*_1_, *b*_1_, *s*_1_), *p*_2_: (*a*_2_, *b*_2_, *s*_2_), …*p*_m_: (*a*_m_, *b*_m_, *s*_m_)} GS*_i_*=grid search (C*_i_*, Param*_i_*, K-CV) where *p_i_* denotes the hyper-parameter, *a*_m_ and *b*_m_ represent lower limit value and upper limit value for *p_i_, s*_m_ is the step length for grid search. 3. Train the primary classifiers with paired hyper-parameters and evaluate the performance of each model by K-CV. Then, select the best models and corresponding hyper-parameters with the highest accuracy. **for***i*= 1, 2, …, *I***do** **for***n* = 1, 2, …, *N***do** C*_i_*′=fit(GS*_i_*(*x_n_*)) C*_i_*′′=high_acc(C*_i_*′) **end for** **end for** 4. Train the best primary classifiers and obtain the classification results. **for***i*= 1, 2, …, *I***do** *q_i_*= C*_i_*′′(*D*); *D*′ = ∅; **for***n* = 1, 2, …, *N***do** **for***i*= 1, 2, …, *I***do** *c_ni_* = *q_i_*(*x_n_*); **end for** *D*′ = *D*′∪((*c_n_*_1_, *c_n_*_2_,…,*c_nI_*,), *y_n_*) **end for** 5. Train the meta-classifier and predict the geological characteristics *q*′ = C(*D*')) 6. **Output:** the prediction results: *Q*(*x*) = *q*′(*q*_1_(*x*), *q*_2_(*x*),…, *q_I_*(*x*))

The SCA-GS prediction model for geological characteristics was developed by Python program. Readers can contact the author to apply for the source program.

## CRediT Author Statement

**Tao Yan:** Calculation, Data curation, Methodology, Investigation, Writing-Original draft preparation, Writing - review & editing.

## Ethics Statement

The authors declare that this work does not involve the use of human subjects or experimentation with animals.

## Declaration of Competing Interest

The authors declare that they have no known competing financial interests or personal relationships that could have appeared to influence the work reported in this paper.

## Data Availability

Data on prediction of geological characteristics during shield tunnelling in mixed soil and rock ground (Original data).
